# Scaling up pre-exposure prophylaxis: A global analysis of processes and challenges regarding the implementation of the pre-exposure prophylaxis guidelines

**DOI:** 10.4102/curationis.v49i1.2817

**Published:** 2026-04-13

**Authors:** Junior M. Ntimani, Andile G. Mokoena-de Beer, Deliwe R. Phetlhu

**Affiliations:** 1 Department of Nursing Science, Faculty of Health Sciences, Sefako Makgatho Health Sciences University, Pretoria, South Africa

**Keywords:** PrEP, HIV, healthcare provider, HIV prevention, processes, challenges

## Abstract

**Background:**

Pre-exposure prophylaxis (PrEP) guideline implementation varies widely across countries as a result of differences in health systems, resources, and HIV priorities. Since 2016, oral PrEP has been available to key populations, yet limited evidence exists on how implementation challenges differ across contexts.

**Objectives:**

This review aimed to explore the processes and challenges in the implementation of PrEP guidelines globally.

**Method:**

An integrative review method was used by searching electronic databases, including PubMed, Medline, EBSCOhost, CINAHL, and Google Scholar, to extract the data. We included qualitative, quantitative, and mixed-method studies relevant to the global implementation of PrEP published in peer-reviewed English journals from 2019 through 2025.

**Results:**

This study emphasises that worldwide PrEP implementation is complex and adaptable, involving progressive provider engagement, task-shifting, and health system integration. Stigma and misunderstandings about PrEP, including structural barriers such as a lack of coverage recognition and supply chain difficulties at odds with the maintenance phase needed to scale up availability, are challenges.

**Conclusion:**

Progress on global PrEP coverage has been blunted by persisting challenges in dismantling structural, financial, and geopolitical blockades to universal access. The model will work if it is implemented with community outreach and creative service delivery, including education, training, and outreach. Structural inequalities need to be considered and crisis-affected communities prioritised to ensure that access is equitable and sustainable. Implemented at scale, PrEP could be a game changer for HIV prevention globally, especially in high-burden countries.

**Contribution:**

This study contributes by synthesising evidence on global PrEP guideline implementation, highlighting health system, structural, and societal challenges.

## Introduction

Africa has made notable progress in HIV testing and treatment. However, primary prevention remains inadequate, underscoring the need to scale up effective strategies such as oral pre-exposure prophylaxis (PrEP), consistent condom use, and targeted behavioural interventions. In 2024, an estimated 1.3 million people were newly infected with HIV globally, with East, Southern, and West-Central Africa continuing to shoulder a substantial share of new infections (UNAIDS 2025). Despite significant progress in testing and treatment, prevention efforts in these regions remain inadequate as a result of persistent structural barriers, limited health system capacity, and stigma affecting key populations (Chimoyi et al. 2022). Underinvestment in prevention programmes and uneven integration of oral PrEP into national responses further constrain impact. To meet the Joint United Nations Programme on HIV and AIDS (UNAIDS) goal of fewer than 200 000 new annual HIV infections globally by 2030 necessitates a significant decrease in new infections (UNAIDS 2025). The expansion of established HIV-prevention strategies, which include broad access to and utilisation of oral PrEP, enhancement of health systems, and the resolution of social and structural obstacles that impede effective implementation, plays an important role in achieving this goal.

Daily oral HIV PrEP serves as a biological preventive intervention for individuals at heightened risk of HIV acquisition. In 2023, over 3.5 million individuals accessed PrEP at least once. More than 75% of this population, totalling 2.6 million, resided in the African region (UNAIDS 2024). The increase in individuals receiving PrEP by 35% from 2022 to 2023 remains insufficient, as the figures fall short of the goal of 10 million users by 2025. Inadequate adoption of PrEP is indicative of a multifaceted array of barriers functioning across various levels. At the individual level, limited awareness, perceived stigma, and concerns regarding side effects frequently restrict the willingness to initiate or adhere to PrEP. At the interpersonal level, insufficient partner support and apprehension regarding relationship conflict may hinder usage. At the community level, existing social norms, misinformation, and stigma regarding key populations diminish demand and acceptance.

At the structural level, national policies concerning PrEP prioritisation have profound implications for availability, accessibility, and service delivery models. Different implementation approaches have been adopted across countries, reflecting variations in epidemiological profiles, health system capacity, and government structures. South Africa’s national PrEP programme illustrates this approach through integration into primary healthcare, nurse-led initiation, and alignment with the Universal Test and Treat strategy, enabling scale-up via routine service platforms (Eakle et al. 2019; National Department of Health [NDoH] 2023). Similarly, Kenya, one of Africa’s earliest adopters, achieved rapid PrEP expansion through differentiated delivery models, strong NGO partnerships, targeted key population clinics, and substantial national training investments (Irungu & Baeten 2020; Ministry of Health Kenya 2022). In contrast, the United States employs a decentralised, insurance-based model supported by federal initiatives such as Ready, Set, PrEP, and community-based dispensing to broaden access (CDC 2021; Siegler et al. 2021). Collectively, these examples demonstrate how national policies, resources, and health system structures shape context-specific PrEP delivery pathways globally.

In 2012, the World Health Organization (WHO) initially recommended PrEP for specific population groups such as female sex workers, men who have sex with men, people who inject drugs, transgender people, people in prisons, and HIV-serodiscordant couples (Sullivan et al. 2019). Then, in 2015, the WHO recommended the use of PrEP for individuals with a substantial risk of contracting HIV. Following the WHO’s endorsement of PrEP, South Africa and other African countries implemented this HIV-preventive strategy beginning in 2016. In 2022, WHO guidance was amended to advocate a client-centred approach that considers individual choice, local contextual circumstances, individual behaviours, and partner traits when assessing who may benefit from PrEP (WHO 2022a).

Several African nations, including Zimbabwe, Zambia, Kenya, Uganda, Nigeria, and Ghana, have implemented oral PrEP programmes through their national public health systems, each employing distinct strategies. Kenya and Zimbabwe have incorporated PrEP delivery into HIV treatment and reproductive health services. Zambia has emphasised community-based PrEP distribution, whereas Ghana and Nigeria are enhancing PrEP access, using demonstration projects aimed at key populations (WHO 2022b). These countries have implemented ambitious national plans to deliver oral PrEP as part of their overall package of HIV-prevention services, with the aim of both decreased new HIV infections and long-term public health benefits. The utilisation of oral PrEP varies globally, significantly shaped by factors including national HIV prevalence, health system capacity, funding availability, policy priorities, community awareness, and stigma levels. Most countries have adopted comparable strategies regarding guidelines and service delivery models to facilitate effective implementation (UNAIDS 2023; WHO 2022a).

## Aim of the study

This study analyses the global implementation processes aiming to better understand international variations in the implementation of PrEP guidelines and the challenges encountered at each stage, especially considering developing global directives and competing goals. Using this evidence is crucial to informing future work on how to improve the delivery of PrEP and accelerate progress towards global HIV-prevention goals.

## Research methods and design

An integrative review design was selected as it facilitates the inclusion and synthesis of various empirical evidence, encompassing qualitative, quantitative, and mixed-methods studies, to produce a comprehensive understanding of the processes and challenges associated with global PrEP guideline implementation (Whittemore & Knafl 2005). The integrative approach differs from systematic or scoping reviews by embracing methodological diversity, making the approach especially appropriate for complex public health issues that encompass policy, health systems, and behavioural aspects. This design facilitated the synthesis of evidence from various contexts to guide future implementation strategies.

### Stage 1: Problem identification

This review was guided by the research question: what are the processes and challenges involved in the global implementation of PrEP guidelines? The question was formulated to ensure clarity and relevance, using the Population, Intervention, Comparison, Outcomes (PICO) framework (eds. Higgins & Green 2013). The Population (P) comprised healthcare providers implementing PrEP guidelines globally and PrEP users. The Intervention (I) focused on the application of national PrEP guidelines, delivery models, strategies, and operational processes for integrating PrEP into routine services. A formal Comparison (C) was not applicable, as the review synthesised qualitative evidence rather than comparing interventions, although implementation differences across health systems were examined. The Outcomes (O) included the identification of implementation processes, facilitators, barriers, and broader system-level challenges.

### Stage 2: Literature search

A literature search was performed between January and June 2025 on PubMed, Medline, EBSCOhost, CINAHL, and Google Scholar. To perform the search, the strategy used the following Boolean operators and used key terms: (‘pre-exposure prophylaxis’ OR ‘PrEP’) AND (‘implementation’ OR ‘processes’ OR ‘delivery’ OR ‘scale-up’) AND (‘barriers’ OR ‘challenges’ OR ‘facilitators’ OR ‘strategies’). Synonyms and related terms were identified by using Medical Subject Headings. We included studies published from 2019 to 2025 to capture recent evidence following the WHO’s 2016 recommendation to scale up PrEP and the subsequent development of national implementation models. The grey literature was not included in this review, and the review is restricted to peer-reviewed empirical studies for methodological rigour. Moreover, ancestry was conducted by using backward and forward citation tracking to identify additional eligible studies.

### Stage 3: Data evaluation

**FIGURE 1:**
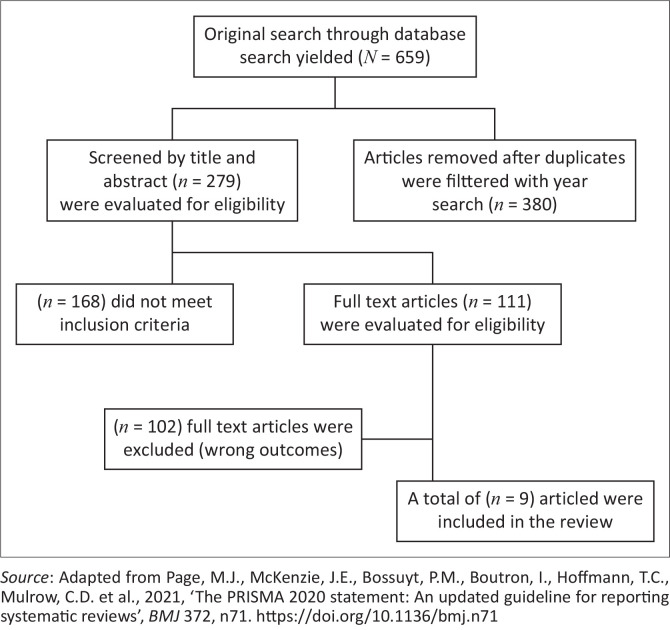
Preferred Reporting Items for Systematic Reviews and Meta-Analyses (PRISMA) flow diagram of study selection.

After searching for eligibility, 111 full-text articles were assessed for inclusion, of which 102 were excluded as not meeting the inclusion criteria. the study selection process is illustrated in figure 1. Despite the numerous quantitative and mixed-methods studies identified and appraised by using quantitative critical appraisal checklists (Joanna Briggs Institute 2017), none met the inclusion requirements in the final synthesis, so no quantitative quality appraisal was carried out. All included studies were qualitative and assessed by the Qualitative Assessment and Review Instrument (QARI) checklist (Joanna Briggs Institute 2017). The nine studies that met the inclusion criteria obtained QARI scores ranging from 6 to 8 out of 10, reflecting aim clarity, methodological rigour, data collection adequacy, participant representation, depth analysis, credibility, and ethical issues. For methodological transparency and quality assurance, a rigorous QARI appraisal is covered in Table 1.

**TABLE 1:** Critical appraisal of included studies across methodological domains using JBI-Qualitative Assessment and Review Instrument.

Author (Year)	Country	Study design	Clear statement of aims	Appropriate methodology	Data collection adequate	Representation of participants	Data analysis rigour	Credibility and/or validity	Philosophical alignment	Influence of the researcher	Logical flow from analysis to interpretation	Ethical considerations	Overall QARI score/10
Baron et al. (2024)	South Africa	Qualitative	✓	✓	✓	✓	✓	✓	✗	✓	✓	✓	9/10
Irungu et al. (2021)	Kenya	Qualitative	✓	✓	✓	✓	✓	✓	✗	✗	✓	✓	8/10
Hicks et al. (2023)	Kenya	Qualitative	✓	✓	✓	✓	✓	✓	✓	✗	✓	✓	9/10
Vanhamel et al. (2023)	Belgium	Qualitative	✓	✓	✓	✓	✓	✓	✗	✗	✓	✓	8/10
Gombe et al. (2024)	Zimbabwe	Qualitative	✓	✓	✓	✗	✓	✓	✓	✓	✓	✓	9/10
Barnighausen et al. (2025)	Eswatini	Qualitative	✓	✓	✓	✓	✓	✓	✓	✗	✓	✓	9/10
Atkins et al. (2022)	Kenya	Qualitative process evaluation	✓	✓	✓	✓	✓	✓	✗	✗	✓	✓	8/10
Smith et al. (2024)	Australia	Qualitative	✓	✓	✓	✓	✓	✓	✓	✗	✓	✓	9/10
Nakambale et al. (2023)	Kenya	Qualitative	✓	✓	✓	✓	✓	✓	✗	✗	✓	✓	8/10

Note: Please see the full reference list of the article, Ntimani, J.M., Mokoena-de Beer, A.G., & Phetlhu, D.R., 2026, ‘Scaling up pre-exposure prophylaxis: A global analysis of processes and challenges regarding the implementation of the pre-exposure prophylaxis guidelines’, Curationis 49(1), a2817. https://doi.org/10.4102/curationis.v49i1.2817, for more information.

JBI, Joanna Briggs Institute; QARI, Qualitative Assessment and Review Instrument.

### Stage 4: Data analysis

Thematic synthesis was conducted in accordance with the framework established by Whittemore and Knafl (2005). Data from eligible studies were compiled into a summary matrix that includes study design, population, setting, processes, challenges, and recommendations. The inductive thematic analysis was performed in four stages: identification of main findings through data reduction, tabulation of extracted data for data display, comparative analysis of data across studies, drawing of conclusions, and verification of themes. Themes were systematically refined through iterative review by all authors until a consensus was achieved, culminating in five overarching themes and their corresponding sub-themes, as detailed in Table 3.

### Stage 5: Presentation of findings

The findings are organised thematically to demonstrate patterns and variations in the implementation of PrEP across various health system contexts. The final synthesis combines descriptive insights regarding processes with analytical insights concerning barriers and facilitators to guide future programme and policy development.

### Ethical considerations

This study was approved by Sefako Makgatho Health Science University Research Ethics Committee (SMUREC) (No. SMUREC/H/485/2023: PG). The review did not involve any participants; thus, informed consent was not obtained. The study was conducted in accordance with the principles of honesty and transparency in the application of the methodology’s phases and accurate data reporting as outlined by Prager et al. (2019).

## Results

### Study demographics

Of the nine articles included, seven reported data from a total of 338 participants, comprising mainly nurse clinicians, counsellors, pharmacists, physicians, and expert clients (Table 2), and two did not specify the exact number of participants. All studies employed a qualitative research approach and were conducted in Australia (1), Belgium (1), Eswatini (1), Kenya (4), South Africa (1), and Zimbabwe (1).

**TABLE 2:** A summary of included studies: Analysis of processes and challenges in the implementation of pre-exposure prophylaxis guidelines globally.

Author and/or country	Population	Approach and/or design and/or methods	Results	Limitations
A1: Barnighausen et al. (2025) Eswatini	**Sample and technique:** Purposive sampling, Healthcare Workers involved with PrEP initiation at any stage-Nurses (54), Mentor mothers, NGO counsellors, Nursing assistants, and expert clients. All were trained in the PrEP framework package implementation.	Qualitative study approach. Setting-Rural and Semi-Urban, Eswatini, Nurse-Led Primary care setting. Free at the point of care. **Interventions:** Development of the framework by Ministry of Health (MoH) and stakeholders, including HCW **Clinic-based routine morning health education for ALL:** Complete a MoH-designed HIV risk-screening tool and to undergo routine HIV testing Positive HIV test = linkage to HIV care and treatment services. Negative HIV test = Eswatini’s standard comprehensive HIV-prevention package, including HIV-prevention counselling, promotion of and free access to condoms, sexually transmitted infection testing, and treatment and further information about PrEP Initiation of PrEP to volunteers and identified at-risk clients. Implementation and support from research entities and universities outside Eswatini **Design:** Qualitative-2 phased semi-structured interviews with HCW, using a standardised interview guide **Analysis:** Inductive analysis using grounded theory tenets	**Process:** **Introduction of PrEP in the country-** Approval of the prevention tool. Improvement in counselling skills. Concerned with the decline in condom use, increase in pregnancy and sexually transmitted infections, and fear of resistance as a result of non-adherence. Insufficient knowledge shared during Health Education HCW. adaptation of the implementation of the guideline alongside existing TB testing. **Challenges:** Poor adoption as a result of the eligibility criterion. Similarity of PrEP pill to HIV + treatment. Blood collection days’ negative impact. Impact of male partners and community members. No extension of PrEP awareness creation to communities. Patriarchy where information is delivered by women HCW. **Recommendations:** All clients to be offered PrEP. Information on more training on the perceived impact of PrEP on health. Use of body weight and risk rather than age as an eligibility criterion. Increase in service points. Involvement and influence of community leaders. Men as targets for awareness. Increase in the number of trained HCW. Use of pamphlets and branded clothing to initiate community engagements.	Certain constructs of the adopted framework were not investigated, particularly the HWC self-efficacy and the implementation environment.
A2: Gombe et al. (2024) Zimbabwe	Purposive sampling. General population clients at high risk for HIV in Zimbabwe. Participants included clients attending one urban family planning clinic and one rural youth centre. A total of 150 HIV-negative clients offered PrEP during pilot intervention (Jan–Jun 2018). Sixty semi-structured interviews conducted. Participants largely serodiscordant couples (SDCs), transactional and/or commercial sex workers (CSWs), and those with partners of unknown HIV status. Age range: 18–49.	Qualitative study approach. Semi-structured in-depth interviews conducted post first and/or second follow-up visits or after PrEP decline. **Data collection:** Took place in public sector facilities, integrating PrEP into existing HIV-prevention services (family planning and youth centres). Participants are categorised into five groups based on acceptance and retention status. **Analysis:** Thematic analysis based on the Health Belief Model constructs: perceived susceptibility, severity, benefits, barriers, cues to action, self-efficacy Coding and analysis performed by a team with iterative codebook development and use of constant comparative methods.	**Process:** High perceived HIV risk: Main driver for uptake, especially among SDCs, CSWs, and those suspecting partner infidelity. Desire to protect self and family from HIV and maintain economic productivity. Feeling empowered and secure when managing HIV prevention. Impact of supportive partners and family members. Integration of PrEP into daily routines. **Challenges:** Fear of being burdened by daily medication or side effects. Fear of non-acceptance and negative responses by partners. Erroneous understanding of how to take PrEP, especially as intermittent use, because of potential risks Logistical matters: transport costs, PrEP-like pills that are taken to treat HIV and/or AIDS. -Interrupted regimen as a result of travel, work, and missing pills Some partners object to the medicine, and in a few cases, people have stopped using it because of partner secrecy. **Recommendations:** Enhance HCW training to improve client counselling on PrEP use, adherence, side effects, and partner communication. Clarify misunderstandings on the administration and persistence of PrEP. Expand PrEP service points to overcome access barriers. Engage male partners and community leaders to increase awareness and acceptance. Integrate PrEP promotion with other reproductive health services Supply information in indigenous languages about the advantages of PrEP	Recruitment from only one urban family planning clinic and one rural youth centre restricts the representativeness of participants, potentially excluding other settings where PrEP uptake may differ.
A3: Smith et al. (2024) Australia	Purposive sampling. Chosen to reflect a variety of provider positions and clinic types. HIV PrEP providers in two Australian jurisdictions. Participants: Twenty-eight healthcare professionals, including general practitioners, sexual health nurses, and sexual health doctors. All participants actively involved in prescribing or providing PrEP after PrEP became publicly subsidised in 2018.	Qualitative study using semi-structured interviews. Interviews conducted in 2019–2020 to capture views after public subsidy introduction for PrEP in Australia. **Analysis:** Thematic analysis applied to identify challenges encountered by clinicians in PrEP provision. Data focused on experiences, perceptions, and practical barriers from the clinicians’ perspective.	**Process:** Nurse-led PrEP delivery was restricted by regulatory and policy restrictions, despite nurses showing competence in earlier PrEP trials. Some clinicians felt frustrated at the underutilisation of nurses in PrEP prescribing and management. Expansion of nurse roles, to improve access and share clinical workload. Equity sought financial access for eligible patients to PrEP (those with Medicare). Patients without medicare (non-citizens, some migrants) face financial and access barriers. General practitioners found it challenging to integrate PrEP into routine consultations, although they saw PRP as quite easy to prescribe. Conflicting clinical demands and a time-limited quality of GPs at consultations hinder PrEP provision being systematic. GPs felt less confident in managing PrEP patients than sexual health nurses and specialists. **Challenges:** Specific providers noted no confidence in advising or managing on-demand PrEP regimens. Complexity of discussing intermittent dosing, which the healthcare provider believes makes counselling more difficult, uncertainty about guidelines, and concerns about adherence. Clinic workload and administrative burden for PrEP provision Ensuring consistent and up-to-date provider knowledge and training. **Recommendations:** Guidance for creating general practice-friendly PrEP guidelines integration. Conduct the advocacy to permit nurse-led PrEP prescribing to reduce wastage in the workforce and improve functional efficiency. A lack of PrEP support opportunities for non-medicare patients without financial hardship. Need for training and measures to incentivise the pros.	Results are reported from the views and context of healthcare providers providing PrEP only. This excludes the barriers or facilitators related to patient or additional stakeholder perspectives that may be omitted from patient experiences.
A4: Vanhamel et al. (2023) Belgium	Purposive sampling of physicians, PrEP nurses, psychologists, and other clinic staff in Belgium, who provide PrEP care to participants from eight HIV clinics across Belgium, for which PrEP has been centralised. Physiotherapists included in the survey allowed for follow-up interviews with a random sample of physiotherapists and an additional survey up-sampling to ensure adequate experiences from different geographic and practice settings. With around 50 h of additional observation in healthcare settings and clinical interactions	Qualitative multiple case study design January 2021 – May 2022 Data collected with semi-structured interviews and ethnographic observations of PrEP implementation practices and adaptations analysis. **Analysis:** Thematic analysis was guided by an adapted form of Extended Normalisation Process Theory, focusing on the following themes: providers adapt practices to local context and constraints.	**Process:** Highlights that the main challenge concerning PrEP service delivery in Belgium is its centralised approach, with special clinics being responsible for the provision of PrEP. Workloads that increased with personalised patient care. Clinics reorganised internal structures and work processes to make PrEP care more efficient without sacrificing quality of care. Providers modified prescribing practices and reallocated roles among staff to align with policies and norms for PrEP delivery, thereby enhancing patient-centred care. Significant task-shifting from physicians to nurses observed, with nurses engaged in increased PrEP delivery activities. Increased collaboration among clinicians treating HIV and the growing team of healthcare workers practising in the field. At times, adaptations were not wholly congruent with official reimbursement rules and guidelines. The system was strained and ‘short-staffed’, laying bare the policy–practice tension. Providers demonstrated considerable adaptive environments through the establishment of multidisciplinary entry points that support PrEP. The adjustments were designed to accommodate an ever-rising workload and to respond to everyone better. **Challenges:** Many providers have found it challenging to strike a balance between service delivery and ensuring attendance, which puts demand on clinic volume. There are a few local clinical adaptations and task-shifting (e.g. nurses’ roles regarding PrEP) rules and policies as written by reimbursement officials, which in turn caused friction between practice and policy frameworks. Providers were constrained by the rigid organisational contexts in the speed and manner by how adaptive changes are introduced. Meeting the psychosocial and nonmedical needs of PrEP users was a necessary but not sufficient condition for responding comprehensively to those at risk of HIV infection. **Recommendations:** Local clinics are requested to adapt the guidelines to their specific needs, and for this reason, incorporate greater flexibility in policy and clinical guidelines, advancing PrEP service innovation. Facilitating and institutionalising task-shifting to nurses, and integration across disciplines, improving service quality and appropriateness.	Some adaptive interventions that were adjunctive or complementary did not fully align with existing reimbursement and regulatory frameworks; findings reflect a shootout between categorical and practice-driven postgraduate medical training in Belgium. Transferability
A5: Hicks et al. (2023) Kenya	Purposive sampling of pregnant and breastfeeding women going to Kenyan maternal and child health (MCH) clinics for both antenatal, prenatal care, and postnatal services. Local staff provide prevention of mother-to-child transmission as well as scarce structural regulations. The target population is pregnant and postpartum women at high risk for HIV infection.	**Qualitative study design:** The Service Accessibility and Readiness Inventory of MCH clinics was conducted in Kenya to assess the capacity of services available there. The focus was on identifying barriers and instigators that facilitate the incorporation of PrEP into routine MCH. The approach was grounded in implementation science, which could inform scale-up strategies.	**Process:** Integration of PrEP into antenatal care (ANC) services at the sites of investigation is acceptable and feasible. Significant facilitators were support from harmonised national policies, prescribing nurses and laying HIV counsellors available; visits for PrEP were coordinated with existing MCH services. **Challenges:** Lack of training of healthcare workers on PrEP service delivery leads to inadequate knowledge of PrEP. Lack of essential forms, stockouts of PrEP medication, and limited HIV space and testing policies for postnatal continuation. **Recommendations:** Task-shifting with no clinical training alleviates burden on both workers and providers. Amending national guidelines to define and possibly expand who is eligible to receive PrEP might even mean more availability for clinics overall. Strengthen healthcare provider training and mentorship through ongoing PrEP delivery in MCH settings. Integration of PrEP delivery systems with adolescent-friendly services and community mobilisation enhances accessibility and long-term retention. Medication and tools for documentation enshrining ensure a consistent supply. Fast-track and group counselling together offer differentiated options for PrEP delivery.	Some of the study settings were previously engaged in PrEP-related research or programs, resulting in a higher level of initial service readiness than in facilities without such involvement. This discrepancy might bias the results and could overestimate the national readiness for PrEP integration. During the survey, many facilities were in the process of changing from paper-based record keeping to electronic management systems. Past data collection may have influenced the availability of paper commodities surveyed (e.g. PrEP cards), so uniform measurements were difficult to gather.
A6: Nakambale et al. (2023) Kenya	Purposive sampling of pharmacy providers at five private pharmacies in Kenya. Clients at risk of HIV acquisition seek PrEP services through these pharmacies	**Qualitative study design:** Pilot implementation of pharmacy-delivered PrEP services charging a fee (~$3 per visit). Providers trained on PrEP initiation and/or continuation using a prescribing checklist with remote clinician oversight. Weekly structured observation reports by research assistants over 6 months (November 2020 – May 2021). Content analysis of 74 observation reports to identify multi-level barriers and strategies. Framework for analysis: Consolidated Framework for Implementation Research (CFIR) domains	**Process:** Community of practice: A non-partner and four partners working on advocacy, policy, research, programme implementation, and commerce to jointly create and design the pharmacy-based PrEP delivery model. The Kenya Engagement with the Ministry of Health and National AIDS & STI Control Program to align the national strategies and policies as a model. A stakeholder consultation agreed on the delivery model, reconfiguring current materials and creating prescribing checklists for pharmacy providers. Local pharmacists in private pharmacies had been taught to initiate and follow everybody on PrEP the same way. **Challenges:** Barrier to PrEP delivery by traditional pharmacies was the need for charging a fee. Reluctant to bring up sensitive subjects such as sexual behaviours and HIV testing with pharmacists. PrEP service delivery was time-consuming and different from other pharmacy services; it also disrupted the normal mode of operation. Providers found it challenging to meet their daily work targets under these conditions. Their attitudes towards it as professionals were also influenced by stigma and personal feelings affecting implementation. As the pharmacy began offering PrEP Services, its workforce entered a period of adjustment and was under pressure to work. **Recommendations:** Implement self-screening tools for clients to assess behavioural HIV risk to reduce discomfort and increase efficiency in pharmacy settings. Flexible appointment scheduling to accommodate pharmacy workflow and client convenience, helping to reduce disruptions in service delivery. Provide frequent and persistent PrEP education to pharmacy staff, particularly those newly hired, to ensure continuous quality and consistency in PrEP delivery. Target the issues associated with financial restrictions, including discussions on affordability and potential subsidy or cost-reduction practices, as client fees were identified as a real impediment. Support provider engagement and reduce stigma by sensitising pharmacy staff to concerns about stigma and hesitancy related to PrEP, emphasising its role in HIV prevention without moral judgments. Actively employ routine programmatic data to track bottlenecks and progress in implementation, enabling decisions to be made for midcourse corrections and troubleshooting. Improved integration with national HIV programs and policies, for supply chain security, regulatory assistance, and to ensure consistency with public health plans.	The research was limited as it focused on the implementation challenges and adjustments early in the innovation process and might not adequately capture long-term sustainability or changing solutions. Observations were obtained from weekly structured observation reports completed by research assistants instead of being direct. Clients or providers relied on qualitative interviews, which may preclude depth of understanding at the individual level.
A7: Baron et al. (2024) South Africa	Purposive sample: adolescent girls and young women (AGYW), a high-risk population for HIV infection in rural South Africa. Qualitative data based on in-depth interviews with five young women, 11 healthcare providers, and four key informant stakeholders who were recruited from a network of organisations through snowball sampling: conducted between January 2021 and January 2022. The context was low-resource clinics integrated within larger health systems and public health problems.	Qualitative study design using CFIR, this study explored multi-level factors related to five domains: Intervention Characteristics, Outer Setting, Inner Setting, the Characteristics of Individuals and Process. In-depth qualitative interviews were conducted with key stakeholders: 5 AGYW with PrEP experience, 11 healthcare providers, and 4 key informants. **Analysis:** Thematic qualitative analysis of coded transcripts based on the CFIR.	**Process:** Initial planning was hampered by erratic and fluctuating funding from the National Department of Health, which delayed vital operational work, leading to communications being fragmented and inconsistent. Multiple stakeholders, such as clinics, national health authorities, and NGOs, were involved, even though coordination was not so good. The fragmented planning, involvement, and execution processes across different actors have priorities. Operational planning and models to deliver PrEP services varied by clinic. There were issues with limiting privacy as staff numbers and spaces in clinics for confidential consultations also limited how they could implement it. **Challenges:** Weak or disjointed monitoring processes limit the ability to assess them in concert with PrEP service outcomes. Few information-sharing platforms between the National Department of Health. Clinics and partners are not allowed to share the monitoring data and feedback in real time. **Recommendations:** Stakeholders pinpointed areas for strengthening monitoring systems to facilitate the functioning of the programme adjustments. Sustainment and Continuous Improvement of PrEP initiation and maintenance protocols were found to be cumbersome by many clinics. Improved systems communication and dissemination and higher quality clinic-level leadership for scale-up.	Efforts targeted implementation processes and system-level limitations rather than outcome measures of client treatment adherence, retention, or HIV incidence measures.
A8: Atkins et al. (2022), Kenya	Purposive sampling comprising 104 participants. 48 PrEP service providers (nurses, clinical officers, HIV peers’ educators, and 12 programme and county managers. Participants recruited from Kiambu, Kisumu, Kisii, Machakos, Migori Mombasa and Nairobi (seven counties).	Qualitative study design with in-depth interviews, which included 12 key informant interviews and 9 focus group discussions in a qualitative study. Data collection between 2017 and 2019. The framework analysis was guided by Kenya’s 2017 National PrEP Implementation Framework, which articulates the approach to PrEP implementation in Kenya. PrEp implementation Institutionalisation. Seven Health System-Level Domains for Thematic Analysis.	**Process:** PrEP rollout, especially in a population of students, without FDA approval. Strong leadership, communication and coordination across on-campus departments and workflows. They reportedly stressed streamlining data collection and allocating resources efficiently. DICEs, community outreaches, sexual and reproductive health clinics. **Challenges:** The shortage of staff, hardening and fragmented incentives in a health system. Modifications included quick step-ups for clients, on-the-job training by peers, extended refill time, and integration of PrEP topics into routine health talks. Stigma keeps people falling off the system, and resolving this challenge requires raising community awareness and dissemination of the information to the targeted population. **Recommendations:** Increasing the prioritisation of PrEP by institutional bodies and extended training for everybody, including the non-to-all levels, support for programs to collaborate on work more effectively.	The framework analysis used was highly deductive, based on the Kenya PrEP. Implementation Framework, which may have precluded identifying additional themes not included in the framework.
A9: Irungu et al. (2021), Kenya	Purposive sampling of 36 health providers interviewed from 25 public HIV care clinics. Providers included clinical officers, nurses, HIV counsellors, social workers, data clerks, and pharmacists. Clinics located in western and central Kenya.	Qualitative process evaluation within the Partners Scale-Up Project. Semi-structured interviews and technical assistance (TA) reports analysed. Framework for Reporting Adaptations and Modifications (FRAME) used to categorise adaptations. Data collected from May to December 2018. Mixed methods with qualitative interviews supplemented by data from TA observations	**Process:** The majority did not consistently perform baseline creatinine testing. Clinics made multiple adaptations: inclusion of PrEP topics in health talks, phone reminders, fast-tracking PrEP clients, service outside regular hours, and dispensing of PrEP in clinical rooms. From the clinics that implemented these changes in care, PrEP initiation and/or continuation was higher. Providers implemented strategies to reduce client stigma and waiting times,thereby improving service convenience. Peers training peers on the job increased skilled provider availability. Less frequent refills decreased clinic visits and improved retention **Challenges:** Increased workload, more documentation, and the way to keep the implementations even after rolling them out. Task-sharing support, simplified documentation, and the promotion of client-centred service models were identified as key implementation strategies.	As the study was conducted within a specific network of public HIV care clinics in Kenya, the findings may not be fully generalisable to other settings with different health system characteristics.

Note: Please see the full reference list of the article, Ntimani, J.M., Mokoena-de Beer, A.G., & Phetlhu, D.R., 2026, ‘Scaling up pre-exposure prophylaxis: A global analysis of processes and challenges regarding the implementation of the pre-exposure prophylaxis guidelines’, *Curationis* 49(1), a2817. https://doi.org/10.4102/curationis.v49i1.2817, for more information.

PrEP, pre-exposure prophylaxis; NGO, non-government organisation; PRP, pre-exposure prophylaxis; GPs, general practitioners; DICE, Delivery, Integration, Coordination and Evaluation; HCW, healthcare workers.

### Thematic findings on global pre-exposure prophylaxis guideline implementation processes and challenges

#### A global review of pre-exposure prophylaxis guideline implementation processes and challenges

The analysis yielded five main themes on the global review of PrEP guideline implementation processes and challenges, namely, Provider Perspectives and Implementation Processes, Client-Level Barriers and Sociocultural Factors, Health System and Structural Challenges, Facilitators and Adaptive Strategies, and Contextual and Comparative Insights (Table 3).

**TABLE 3:** Identified themes and sub-themes.

Themes	Sub-themes
1. Provider perspectives and implementation processes	1.1. Initial enthusiasm, evolving concerns and training apps 1.2. Task-shifting and role expansion 1.3. Policy and regulatory barriers 1.4. Implementation workflow challenges
2. Client-level barriers and sociocultural factors	2.1. Stigma and misconceptions 2.2. Communication barriers between clients and providers 2.3. Gender and power dynamics 2.4. Financial barriers
3. Health systems and structural challenges	3.1. Fragmented planning and communication 3.2. Resource shortages 3.3. Workload and capacity constraints 3.4. Monitoring and data systems
4. Facilitators and adaptive strategies	4.1. Task-shifting and multidisciplinary collaboration 4.2. Simplification of procedures 4.3. Community engagement and stigma reduction 4.4. Client-centred innovations 4.5. Ongoing training and support
5. Contextual and comparative insights	5.1. Variation between settings 5.2. Financial and policy contexts 5.3. Adaptability and sustainability considerations

The results of this integrative review are summarised in Table 4, which outlines the main findings, associated challenges, and key facilitators identified across the reviewed studies. A detailed narrative description of each theme follows.

**TABLE 4:** A summary of study’s main findings on global pre-exposure prophylaxis guideline implementation.

Theme	Main findings	Key challenges	Strategies and/or facilitators
1. Provider perspectives and implementation processes	Providers were initially enthusiastic, perceiving PrEP as an effective method for HIV prevention. Task-shifting enhances operational efficiency.	Deficiencies in training, inadequate counselling competencies, regulatory limitations on nurse prescribing, and workflow challenges	Ongoing professional development, effective supervision, and well-defined task-shifting policies
2. Client-level barriers and sociocultural factors	Individual motivation is influenced by perceived HIV risk and the support of partners	Stigma, misconceptions, restricted communication with healthcare providers, gender and power dynamics, and financial barriers	Community sensitisation, partner engagement, privacy protection, and subsidies aimed at reducing out-of-pocket expenses
3. Health system and structural challenges	The integration of PrEP into national programs is characterised by inconsistency	Disjointed planning, inventory shortages, labour deficiencies, and inadequate data systems	Enhanced coordination, sufficient supply chains, and refined monitoring and evaluation frameworks
4. Facilitators and adaptive strategies	Task-shifting, streamlined initiation, and community involvement enhanced accessibility	Resistance to policy modification and constrained sustainability frameworks	Decentralised and differentiated service delivery involves multidisciplinary collaboration and community outreach
5. Contextual and comparative insights	The success of implementation is contingent upon economic, policy, and cultural contexts	Regulatory rigidity in high-income contexts, stigma and resource constraints in low- and middle-income countries	Adaptation of policies, flexible guidelines, and alignment between national and local innovations

PrEP, pre-exposure prophylaxis.

### Theme 1: Provider perspectives and implementation processes

The experience of providers and the processes of implementation appeared to be predominant across studies. The findings pertained to training adequacy, job clarity, task delegation, and counselling capability. This theme was reported in seven of the nine included studies and included the studies from Eswatini, Kenya, South Africa, Belgium, and Australia (Barnighausen et al. 2025; Baron et al. 2024; Gombe et al. 2024; Hicks et al. 2023; Nakambale et al. 2023; Smith et al. 2024; Vanhamel et al. 2023). The results are organised according to the following sub-themes:

#### Sub-theme 1.1: Initial enthusiasm, evolving concerns, and training gaps

Several studies highlighted in the study demonstrated an initial enthusiasm for PrEP by healthcare professionals (Barnighausen et al. 2025; Hicks et al. 2023). This study highlights the leading intention of providers to initiate PrEP as a statistically effective, biomedical HIV-prevention option. With time, several issues arose in relation to PrEP integration into routine service delivery. Concerns of decreased condom use and expanded unplanned pregnancies seem to stem from inadvertent behaviour changes, which is also known as risk compensation, in which people feel safer and adapt their sexual behaviour as a result. These findings are not necessarily the result of PrEP itself but rather reflect the wider shift in risk perception related to PrEP uptake. Additionally, concerns were expressed regarding the potential development of drug-resistant viral strains as a result of inconsistent adherence or incomplete implementation of PrEP protocols, reflecting patterns observed in previous HIV treatment trials. The evolving concerns underscore the need for continued training, support, and provider-optimisable evidence-based methods to maintain provider confidence for future PrEP rollout.

The review found several studies that highlighted continued provider training and clinical deficits in PrEP service delivery (Baron et al. 2024; Gombe et al. 2024). This study noted that insufficient skills in counselling, poor knowledge of PrEP dosing protocols, and uncertainty about how to manage side effects were all critical factors that reduced the quality of service delivery. The limitations diminished provider confidence and undermined effective client engagement. Thus, ongoing training, clinical mentorship, and supportive supervision are essential to enhance provider capacity and ensure the delivery of consistent, high-quality PrEP counselling and management.

#### Sub-theme 1.2: Task-shifting and role expansion

A review of articles tracing the scale-up of PrEP services utilised task-shifting approaches in varied settings (Hicks et al. 2023; Nakambale et al. 2023; Vanhamel et al. 2023). Our results from this review demonstrate that task-shifting PrEP delivery to nurses, lay counsellors, and pharmacy workers increased service availability and addressed clinical bottlenecks. Initiation of PrEP was both triggered and facilitated by healthcare workers offering counselling and adherence support, particularly in resource-poor or highly utilised settings. While these results are encouraging, regulatory and policy barriers emerged as common impediments to full implementation and scale-up of task-shifting.

#### Sub-theme 1.3: Policy and regulatory barriers

This review underscores that regulatory frameworks and policy misalignment features of regulatory architecture are fundamental implementation bottlenecks for PrEP scale-up in varied contexts (Smith et al. 2024; Vanhamel et al. 2023). The results indicate that variations in the scope of practice and prescribing authority significantly impacted the rollout of PrEP. Constraints on prescribing in various settings have restricted qualified personnel, including STI nurses, medical assistants, and harm reduction staff, from initiating PrEP, consequently limiting service access. Organisation-neutral reimbursement policies and funding mechanisms frequently do not cover PrEP services provided outside conventional clinical settings, resulting in financial disincentives for providers and limiting service expansion (Killelea et al. 2022). These structural barriers marginalised the complexity of innovative and restrained uptake of PrEP through non-traditional care models such as pharmacy or community-based mode. A prominent point that kept cropping up was the need for legislative change and delivery systems that are more inclusive and adaptable in terms of bringing PrEP at scale to priority groups.

#### Sub-theme 1.4: Implementation workflow challenges

These included articles indicate that prolonged initiation processes and structural barriers prevented the implementation of PrEP (Nakambale et al. 2023; Barnighausen et al. 2025). The findings of this review also indicate that implementation problems within the workflows affected not only the health service providers but also the clients, often leading to significant delays and frustrations as well as a reduction in service uptake. There was a consensus among providers that documentation increased the burden on their already strained system and implemented very rigid protocols, which led to longer waiting times and many visits for clients. The study results illuminate the importance of streamlining PrEP onboarding steps and operations to better deliver services, improve client satisfaction, and support wider uptake.

### Theme 2: Client-level barriers and sociocultural factors

This review identified client-level barriers and sociocultural factors that influence PrEP uptake and adherence across several studies. Evidence for this theme came from five studies conducted in Kenya, Zimbabwe, Eswatini, and South Africa (Atkins et al. 2022; Baron et al. 2024; Barnighausen et al. 2025; Gombe et al. 2024; Nakambale et al. 2023). Below are the sub-themes that address these challenges.

#### Sub-theme 2.1: Stigma and misconceptions

Two studies that are included in this review underlined stigma as one of the main reasons for a lack of PrEP initiation and non-adherence (Atkins et al. 2022; Gombe et al. 2024). Findings from this review suggest that many clients were concerned with being associated with taking PrEP pills that appeared like antiretroviral therapy, accessing PrEP service, being labelled as having HIV, or being seen as sexually promiscuous. The stigma had been largely perpetuated by persistent, community-wide misconceptions about PrEP: both what it was developed to do and for whom. These dynamics created barriers to uptake, discouraged potential adopters, and adversely impacted adherence. The literature reviewed identifies the need for broad, locally developed, PrEP-normative communication campaigns to counteract misconceptions.

#### Sub-theme 2.2: Communication barriers between clients and providers

The study included in this review showed client discomfort in discussing sexual behaviours with healthcare providers, particularly within the pharmacy-based PrEP delivery models (Nakambale et al. 2023). This finding is in keeping with the observation that a lack of eagerness on the part of providers to discuss openly constrained complete risk evaluation and counselling. The lack of communication compromised providers’ capacity to personalise PrEP communication and adherence support. Client-centred communication enhancements to support discretion, neutrality, and cultural competency may improve the quality of PrEP services.

#### Sub-theme 2.3: Gender and power dynamics

The review also found studies that demonstrate that sociocultural norms – in particular, patriarchal norms – influence PrEP acceptability and use in women (Barnighausen et al. 2025; Baron et al. 2024). Results suggest that PrEP acceptability and continuation were often undermined by a lack of willingness of male partners to support women’s autonomy and control in HIV-prevention decisions. This context constrained women’s capacity to utilise PrEP effectively as an HIV-prevention tool. The literature review underscores the importance of designing and implementing interventions that actively involve male partners, as their support is essential in influencing women’s decisions and autonomy in HIV prevention. Enhancing male partner involvement in conjunction with women’s empowerment can create supportive environments that diminish relational barriers, improve PrEP acceptability, and encourage sustained use among at-risk women.

#### Sub-theme 2.4: Financial barriers

Two studies included in this review mentioned that financial costs associated with PrEP provision, such as charges at pharmacy-based delivery models and poor insurance reimbursement, presented major hindrances to client access (Nakambale et al. 2023; Smith et al. 2024). This review demonstrates the importance of cost in achieving equity in PrEP delivery. Interventions addressing economic barriers, including the removal of user fees, provision of transport subsidies, insurance coverage for PrEP services, and the integration of PrEP into existing publicly funded programmes, are critical elements of comprehensive HIV-prevention strategies. These strategies may decrease out-of-pocket expenses, enhance equitable access, and facilitate sustained engagement among economically disadvantaged and high-risk groups.

### Theme 3: Health system and structural challenges

Multiple studies in this review highlighted health system and structural obstacles to PrEP adoption. Five studies contributed to this theme, including those from Kenya, South Africa, Belgium, and Eswatini (Baron et al. 2024; Hicks et al. 2023; Irungu et al. 2021; Nakambale et al. 2023; Vanhamel et al. 2023). Below are the sub-themes that speak to these challenges.

#### Sub-theme 3.1: Fragmented planning and communication

The review identified that fragmentation in programme design, characterised by misaligned implementation frameworks, overlapping partner activities, and ambiguous role delineation between government and implementing agencies, obstructed coordinated PrEP delivery. Inadequate communication among national authorities, clinics, and implementing partners resulted in inconsistent policy interpretation, duplication of reporting systems, delayed feedback loops, and fragmented data sharing. These issues undermined timely decision-making and the cohesive scale-up of PrEP services (Baron et al. 2024; Irungu et al. 2021). The findings indicate that conflicting priorities and ambiguous targets among stakeholders hindered coordinated efforts, obstructing the development of cohesive and sustainable PrEP programmes.

#### Sub-theme 3.2: Resource shortages

This review identified that the ongoing stockouts of critical resources, such as PrEP drugs, test kits, and stationery, affect service continuity, particularly in rural clinics (Baron et al. 2024; Hicks et al. 2023). These results suggest that the resources that are reportedly inadequate to meet the high demand for service interruptions reduce the trust of patients in PrEP services. Efficient supply chains and sufficient resources are essential for reliable processes managing continuous use of and adherence to PrEP among users.

#### Sub-theme 3.3: Workload and capacity constraints

Three articles in this review found that large volumes of patients coupled with inadequate staffing resulted in excessive workloads for healthcare workers (Irungu et al. 2021; Nakambale et al. 2023; Vanhamel et al. 2023). These pressures, identified as key influences on primary resources for PrEP providers, signal the resultant and decreased time available to undertake important activities such as brief adherence interviews, care engagement activities, or ensured delivery of meaningful client-centred counselling. The need for workforce enhancement and resource allocation becomes more urgent to ensure continuity of PrEP programmes as a result of burnout or loss of staff in the health systems.

#### Sub-theme 3.4: Monitoring and data systems

This review found an article that highlighted the continued deficiencies in monitoring and data management systems, which obfuscate effective oversight of overall programmes and guidance by data (Baron et al. 2024). The findings underscore the critical need for strengthening surveillance and evaluation frameworks to enable timely adaptations and improvements in PrEP service delivery. Robust data systems can facilitate accurate tracking of client outcomes, stock management, and service coverage, which are essential for responsive and sustainable programme implementation. Better performing these systems will also enable better policy decisions and resource allocation to address new challenges in the PrEP implementation scale-up.

### Theme 4: Facilitators and adaptive strategies

Several of the included studies emphasised facilitators and adaptive strategies for enhancing PrEP implementation. These strategies focused on strengthening service delivery through task-shifting, community engagement, simplified procedures, and client-centred innovations. Facilitators and strategies were described in six studies, particularly those from Kenya, Belgium, Eswatini, and South Africa (Barnighausen et al. 2025; Baron et al. 2024; Gombe et al. 2024; Hicks et al. 2023; Nakambale et al. 2023; Vanhamel et al. 2023). The pertinent findings are explored under the following sub-themes.

#### Sub-theme 4.1: Task-shifting and multidisciplinary collaboration

This review identified articles documenting the use of task-shifting as a strategy to expand PrEP service delivery (Hicks et al. 2023; Vanhamel et al. 2023). The findings highlight that adaptive service models combining task-shifting with collaboration among clinicians, nurses, counsellors, and community health workers can significantly improve service efficiency and client outcomes. By redistributing responsibilities, such as PrEP initiation and adherence support, to a broader range of health cadres, these models reduce bottlenecks in clinical settings and extend reach to underserved populations. Task-shifting also supports the sustainability of PrEP programmes by addressing workforce shortages and enhancing provider capacity. Successful implementation of these models necessitates clear role definitions, sufficient training, and supportive supervision to ensure service quality and maintain client trust.

#### Sub-theme 4.2: Simplification of procedures

The review found that simplifying service initiation and broadening eligibility criteria can promote PrEP uptake, improve client convenience, and eliminate barriers to care (Barnighausen et al. 2025; Baron et al. 2024). These findings indicate that simplified clinic procedures, for example, offering same-day initiation of PrEP and loosening eligibility criteria for clients are associated with earlier access and increased re-engagement with care. Streamlined processes facilitate service delivery for providers and decrease client waiting times (Bonett et al. 2024). These efficiencies reduce frustration and enhance overall satisfaction among PrEP users. Furthermore, implementing flexible eligibility criteria that consider individual risk profiles, instead of strict categorical qualifications, improves inclusivity and guarantees that high-risk populations retain access. This approach is particularly important for resource-poor settings, in which the constraint on resources as a result of human-resource limits necessitates streamlining the delivery of services to increase the effectiveness of programmes.

#### Sub-theme 4.3: Community engagement and stigma reduction

This review highlights a study that shows the involvement of community leaders and male partners, along with culturally relevant educational initiatives, significantly decreased stigma and enhanced community acceptance of PrEP (Gombe et al. 2024). The findings underscore the significance of engaging key community leaders and customising communication to align with local cultural contexts to promote supportive environments for PrEP adoption. These approaches effectively address misconceptions, normalise PrEP usage, and promote ongoing adherence within target populations.

#### Sub-theme 4.4: Client-centred innovations

This review identified studies that highlight innovations, including self-screening tools, flexible scheduling options, and the decentralisation of service delivery via pharmacies and telehealth platforms, as effective strategies for addressing client preferences and enhancing PrEP access (Nakambale et al. 2023). These client-centred approaches reduce barriers related to convenience and privacy, thereby fostering greater uptake and adherence. By aligning service delivery with user needs, these innovations contribute to more responsive and accessible PrEP programmes.

#### Sub-theme 4.5: Ongoing training and support

This review highlights studies that emphasise the need for continuous capacity building and refresher providers’ skills as well as quality of PrEP services (Hicks et al. 2023). The findings imply that in-service refresher training and continued supportive supervision are significant to ensure that healthcare providers are equipped with the necessary information, such as guidelines to improve their counselling skills. Continuous education bolsters PrEP provision trust and ensures consistent, good-quality PrEP delivery.

### Theme 5: Contextual and comparative insights

This review synthesised findings from extensive research to identify contextual and comparative insights. The findings signify the influences of different legal issues, economic statuses, and sociocultural factors on the implementation of PrEP programmes. Differences between high-income and low- to middle-income countries affected factors including task-shifting, affordability, policy support, and community engagement. Cross-context insights were drawn from seven studies conducted across high-income and low-income settings, including Australia, Belgium, Kenya, South Africa, and Eswatini (Barnighausen et al. 2025; Baron et al. 2024; Gombe et al. 2024; Hicks et al. 2023; Nakambale et al. 2023; Smith et al. 2024; Vanhamel et al. 2023). The theme is analysed through the following sub-themes.

#### Sub-theme 5.1: Variation between settings

The reviewed articles published previously have shown the variation in different contexts of implementation related to high-income, as well as low-income countries (Barnighausen et al. 2025; Smith et al. 2024; Vanhamel et al. 2023). This research shows that regulatory inflexibility and a narrow scope of practice provide substantial obstacles in developed settings, often limiting provider roles and service breadth. Economic, supply chain, and sociocultural determinants such as stigma and gender norms continue to challenge PrEP introduction into African contexts. Understanding these multiple barriers is important for tailoring interventions tailored to suit the needs of diverse contexts and promote global PrEP equity.

#### Sub-theme 5.2: Financial and policy contexts

This review underscores the economic practicality and the regional variation shaping access to, affordability of, and integration of PrEP within health systems (Nakambale et al. 2023; Smith et al. 2024). The results show that changes in funds as mechanisms, insurance coverage, and reimbursement policies create differences in the availability of services and client engagement. Wider economic and policy issues aside, context-specific funding options are needed to ensure that access to PrEP is equitable, especially for populations that are marginalised or most at risk. In different settings, their bespoke approaches will be essential to overcome the financial challenge and realise sustainable programmatic coverage.

#### Sub-theme 5.3: Adaptability and sustainability considerations

This review identified studies that reinforce consistent messages on the need to synergise national policies with local innovations for the sustainable scale-up of PrEP services (Baron et al. 2024). The findings underscore the necessity of facilitating flexible and context-specific delivery models while maintaining a continuous supply of financial, human, and material resources. This alignment underpins programme resilience, responsiveness to community needs, and the future iteration for PrEP integration into existing health systems. A lack of attention to these factors is likely to be the reason equitable and sustainable PrEP access is so difficult to achieve.

## Discussion

This integrative study explored the global view of the implementation of PrEP. The results reveal continuous challenges together with adaptation strategies necessary for wider scalability and sustainability in diverse settings.

### Provider dynamics and implementation processes

Healthcare providers are essential to the successful adoption of PrEP. Research conducted in South Africa, Kenya, and Eswatini indicates that initial enthusiasm among providers was mitigated by insufficient training, changing clinical issues, and increased administrative responsibilities (Barnighausen et al. 2025; Baron et al. 2024; Hicks et al. 2023). Providers consistently highlighted insufficient counselling skills, uncertainty regarding PrEP dosing, and a lack of confidence in managing side effects, all of which impacted service quality (Baron et al. 2024; Gombe et al. 2024). The findings corroborate existing global evidence indicating that continuous mentorship and standardised training are crucial for maintaining quality and enhancing provider confidence in PrEP service delivery.

Task-shifting has become a prevalent strategy in various contexts, such as Kenya, Belgium, and Eswatini (Hicks et al. 2023; Nakambale et al. 2023; Vanhamel et al. 2023). The delegation of responsibilities to nurses, chemists, and lay counsellors enhanced service accessibility and alleviated bottlenecks, aligning with the WHO’s focus on differentiated service delivery. Several studies have identified regulatory and reimbursement constraints that limit the prescribing authority of nurses and chemists (Smith et al. 2024; Vanhamel et al. 2023). This regulatory rigidity highlights the necessity for legislative flexibility to institutionalise task-shifting and improve PrEP accessibility.

### Client barriers and sociocultural contexts

Stigma, gender norms, and misinformation were identified as significant barriers to PrEP uptake and adherence (Atkins et al. 2022; Gombe et al. 2024). In Kenya and Zimbabwe, the stigma associated with being labelled as HIV-positive or sexually promiscuous deterred clients from starting or maintaining PrEP (Gombe et al. 2024). Pharmacy-based models have shown that discomfort in discussing sexual behaviour with providers negatively impacts adherence and personalised counselling (Nakambale et al. 2023). The observations underscore the significance of privacy protection, culturally competent communication, and stigma-sensitive service environments.

Gendered power dynamics significantly limited women’s utilisation of PrEP, as male partners frequently opposed or dissuaded women’s engagement in prevention initiatives (Barnighausen et al. 2025; Baron et al. 2024). In alignment with research conducted in Kenya and South Africa, the engagement of men and the challenge of patriarchal norms are essential strategies for enhancing women’s autonomy and ensuring sustained PrEP utilisation. Economic barriers, including user fees in private pharmacies and insufficient insurance coverage, were observed in both high- and low-income contexts (Nakambale et al. 2023; Smith et al. 2024). Addressing affordability via subsidies or incorporating into public health systems is essential for ensuring equitable access.

### Health system and structural factors

System-level barriers, such as fragmented planning, resource shortages, and inadequate monitoring systems, were identified in various studies (Baron et al. 2024; Irungu et al. 2021). Inadequate coordination among ministries, clinics, and implementing partners resulted in inconsistent data reporting, delayed feedback, and inefficiencies in the scale-up of PrEP (Baron et al. 2024). Frequent medication stockouts and insufficient staffing in rural facilities compromised service continuity (Baron et al. 2024; Hicks et al. 2023). Workforce shortages and administrative burdens similarly diminished providers’ capacity to deliver high-quality counselling (Nakambale et al. 2023; Vanhamel et al. 2023). The findings indicate that effective scale-up relies on robust supply chains, sufficient human resources, and operational data systems.

### Facilitators and innovations promoting pre-exposure prophylaxis uptake and delivery

Multiple adaptive strategies enabled efficient PrEP delivery. Research conducted in Kenya, Belgium, and Eswatini indicates that task-shifting and multidisciplinary collaboration enhance efficiency and broaden reach (Barnighausen et al. 2025; Hicks et al. 2023; Vanhamel et al. 2023). Simplifying procedures, including same-day initiation, reduced eligibility restrictions, and multi-month dispensing, has improved convenience and retention (Barnighausen et al. 2025; Baron et al. 2024). Interventions for community engagement that include local leaders and male partners have been effective in normalising PrEP use and reducing stigma (Gombe et al. 2024). Client-centred innovations, including self-screening tools, flexible scheduling, and pharmacy-based delivery models, have enhanced privacy and accessibility (Nakambale et al. 2023). Ongoing provider training and mentorship are essential for maintaining quality and programme fidelity (Hicks et al. 2023).

### Contextual and comparative insights

Comparative analyses across contexts indicated that the capacity of health systems, the structures of financing, and the flexibility of policies influence the outcomes of PrEP implementation. Research conducted in high-income contexts such as Belgium and Australia has highlighted restrictive reimbursement policies and constrained nurse prescribing rights (Smith et al. 2024; Vanhamel et al. 2023). Conversely, in sub-Saharan Africa, barriers arise from resource limitations, fragmented governance, and sociocultural resistance (Baron et al. 2024; Hicks et al. 2023). Research from Kenya and Eswatini indicates that decentralised models and community engagement may mitigate these barriers (Barnighausen et al. 2025; Nakambale et al. 2023). The findings highlight the necessity of context-sensitive policy frameworks that reconcile international standards with local adaptability to ensure the global scale-up of PrEP.

### Results summary

The results of this review confirm international trends regarding the operational and contextual difficulties in the uptake of PrEP by various health systems. Gaps in training, limited confidence among providers, and fragmented implementation processes are identified in Kenya, Zimbabwe, and Eswatini studies that obstruct regular PrEP implementation and scale-up (Atkins et al. 2022; Barnighausen et al. 2025; Gombe et al. 2024). Evidence from high-income settings such as Belgium and Australia consistently suggests structural obstacles, including workflow inefficiencies, the burden of documentation, and limited PrEP integration within routine clinical practice (Smith et al. 2024; Vanhamel et al. 2023). In line with this review, international literature highlights the role of sociocultural and behavioural considerations such as stigma, gender dynamics, perceptions of PrEP efficacy, and concerns about risk compensation in uptake and continuation. The facilitators highlighted from this synthesis, including task-shifting, simplified initiation protocols, community engagement, and collaborative partnerships, are generalisable enablers of successful PrEP implementation globally.

The global scale-up of PrEP is making progress but has major barriers to overcome. Achieving its impact on prevention must involve addressing structural, financial, and social constraints while strengthening training, learning, and outreach. Collaborative approaches that use local resources, innovative delivery models, and supportive policies are essential to equitable and sustainable implementation. While PrEP has proven to be an intervention, its potential will go unfulfilled unless action is coordinated and decisive. By addressing systemic inequities, centring affected communities, and scaling adaptive approaches, HIV transmission can be further reduced, enabling high-burden countries to move closer to an HIV-free generation.

### Limitations and future research

The PrEP guidelines in several countries differ and are widely influenced by the practice pattern, such as healthcare infrastructure, fund availability, and sociocultural factors. This diversity restricts the applicability of the findings to varied settings. Additionally, consider the effects of enabling policies, and more effectively direct resource allocation for the scale-up and sustainability of PrEP uptake.

### Implications for policy and practice

#### Strengthening provider training and support

Providers receive ongoing training in delivery, supported by policies that mandate ongoing professional development to ensure timely, confident, and high-quality client engagement.

#### Simplify service delivery and eligibility criteria

Flexible initiation processes and risk-informed eligibility can reduce client and provider burdens. The policies should support the integration of PrEP into the existing sex and reproductive health services provided.

#### Addressing stigma and sociocultural barriers

Tailored strategies are needed to address gender barriers, including culturally informed education and engagement with community gatekeepers and male partners.

#### Enhancing resource allocation and health system strengthening

Effective PrEP care cascades require robust supply chains, an adequate workforce, and strong monitoring systems. Policymakers should prioritise infrastructure and data systems.

#### Increasing financial accessibility

Equitable access to PrEP requires policy frameworks that reduce financial barriers through subsidies or universal insurance coverage.

#### Foster context-sensitive implementation

Policies must be flexible for local adaptation, considering the diversity in regulatory frameworks, cultural practices, and health system performance in different regions.

### Recommendations

For equitable and sustained implementation, a collaborative model that relies on local assets, new delivery models, and enabling policies is crucial. PrEP is a proven intervention, but its full impact will not materialise without audacious, coordinated action. If we tackle systemic inequities, place the affected communities at the centre, and continue scaling adaptive strategies, HIV transmission can be further curtailed, and high-burden countries will be closer to an HIV-free generation.

## Conclusion

Scaling up the global implementation of PrEP guidelines reflects increased commitment to strengthening HIV prevention through policy adoption, health system integration and expanded service delivery models. Achieving the maximum preventive value of PrEP also requires tackling structural, financial, and social constraints while strengthening learning, training, and outreach.
